# Supplementary material for: SARS-CoV-2 antibody trajectories after a single COVID-19 vaccination with and without prior infection
Source: Nat Commun. 2022 Jun 29;13:3748. doi: 10.1038/s41467-022-31495-x (PMC9243074; doi:10.1038/s41467-022-31495-x)
Supplement: Supplementary file 1 — Supplementary Information [file 41467_2022_31495_MOESM1_ESM.pdf]

Supplementary information for

SARS-CoV-2 antibody trajectories after a single COVID-19 vaccination with and without prior infection

Jia Wei, Philippa C. Matthews, Nicole Stoesser, Ian Diamond, Ruth Studley, Emma Rourke, Duncan Cook, John I Bell, John N Newton, Jeremy Farrar, Alison Howarth, Brian D. Marsden, Sarah Hoosdally, E Yvonne Jones, David I Stuart, Derrick W. Crook, Tim E. A. Peto, A. Sarah Walker, David W. Eyre, Koen B. Pouwels and the COVID-19 Infection Survey team

Contents

• Supplementary Tables 1-4

• Supplementary Figures 1-4

	Without prior infection			With prior infection				
	ChAdOx1 (N=70,146)	BNT162b2 (N=47,065)	mRNA-1273 (N=3,591)	ChAdOx1 (N=10,207)	BNT162b2 (N=10,116)	mRNA-1273 (N=807)	Total (N=141,932)	p value
**Age**								< 0.001
**Median**	55	39	35	52	34	32	50	
**Q1, Q3**	46, 64	30, 63	28, 41	44, 61	24, 48	26, 39	37, 63	
**Sex**								< 0.001
**Female**	37,109 (52.9%)	25,469 (54.1%)	1,751 (48.8%)	5,424 (53.1%)	5,466 (54.0%)	374 (46.3%)	75,593 (53.3%)	
**Male**	33,037 (47.1%)	21,596 (45.9%)	1,840 (51.2%)	4,783 (46.9%)	4,650 (46.0%)	433 (53.7%)	66,339 (46.7%)	
**Ethnicity**								< 0.001
**White**	65,757 (93.7%)	43,412 (92.2%)	3,315 (92.3%)	9,333 (91.5%)	8,857 (87.6%)	691 (85.6%)	131,365 (92.6%)	
**Black**	563 (0.8%)	365 (0.8%)	18 (0.5%)	124 (1.2%)	162 (1.6%)	11 (1.4%)	1,243 (0.9%)	
**Asian**	2,366 (3.4%)	1973 (4.2%)	135 (3.7%)	510 (5.0%)	660 (6.5%)	55 (6.8%)	5,699 (4.0%)	
**Mixed**	860 (1.2%)	845 (1.8%)	80 (2.2%)	130 (1.3%)	295 (2.9%)	31 (3.8%)	2,241 (1.6%)	
**Others**	600 (0.9%)	470 (1.0%)	43 (1.2%)	110 (1.1%)	142 (1.4%)	19 (2.4%)	1,384 (1.0%)	
**Household size**								< 0.001
**1**	11,496 (16.4%)	6,679 (14.2%)	406 (11.3%)	1,466 (14.4%)	1,064 (10.5%)	83 (10.3%)	21,194 (14.9%)	
**2**	31,449 (44.8%)	20,430 (43.4%)	1,297 (36.1%)	3,994 (39.1%)	3,586 (35.4%)	276 (34.2%)	61,032 (43.0%)	
**3**	11,660 (16.6%)	8,371 (17.8%)	759 (21.1%)	1,897 (18.6%)	2,064 (20.4%)	162 (20.1%)	24,913 (17.6%)	
**4**	11,297 (16.1%)	7,960 (16.9%)	835 (23.3%)	2,010 (19.7%)	2,193 (21.7%)	194 (24.0%)	24,489 (17.3%)	
**5+**	4,244 (6.1%)	3,625 (7.7%)	294 (8.2%)	840 (8.2%)	1,209 (12.0%)	92 (11.4%)	10,304 (7.3%)	
**Deprivation percentile**								< 0.001
**Median**	62	60	58	59	56	55	61	
**Q1, Q3**	38, 82	36, 81	35, 81	34, 81	31, 78	29, 79	37, 81	
**Region**								< 0.001
**North East**	3,019 (4.3%)	1,790 (3.8%)	105 (2.9%)	450 (4.4%)	426 (4.2%)	33 (4.1%)	5,823 (4.1%)	
**North West**	7,288 (10.4%)	4,921 (10.5%)	326 (9.1%)	1,499 (14.7%)	1,380 (13.6%)	103 (12.8%)	15,517 (10.9%)	
**Yorkshire and the Humber**	6,060 (8.6%)	3,632 (7.7%)	417 (11.6%)	931 (9.1%)	847 (8.4%)	99 (12.3%)	11,986 (8.4%)	
**East Midlands**	4,822 (6.9%)	3,190 (6.8%)	120 (3.3%)	708 (6.9%)	646 (6.4%)	20 (2.5%)	9,506 (6.7%)	
**West Midlands**	5,822 (8.3%)	3,696 (7.9%)	191 (5.3%)	854 (8.4%)	787 (7.8%)	33 (4.1%)	11,383 (8.0%)	

**East of England**	6,671 (9.5%)	4,366 (9.3%)	345 (9.6%)	956 (9.4%)	862 (8.5%)	57 (7.1%)	13,257 (9.3%)
**London**	8,546 (12.2%)	7,932 (16.9%)	765 (21.3%)	2,015 (19.7%)	2,473 (24.4%)	232 (28.7%)	21,963 (15.5%)
**South East**	10,237 (14.6%)	5,926 (12.6%)	567 (15.8%)	1,217 (11.9%)	1,017 (10.1%)	110 (13.6%)	19,074 (13.4%)
**South West**	6,154 (8.8%)	4,075 (8.7%)	313 (8.7%)	513 (5.0%)	542 (5.4%)	44 (5.5%)	11,641 (8.2%)
**Northern Ireland**	1,859 (2.7%)	1,745 (3.7%)	21 (0.6%)	187 (1.8%)	278 (2.7%)	8 (1.0%)	4,098 (2.9%)
**Scotland**	5,861 (8.4%)	3,835 (8.1%)	346 (9.6%)	514 (5.0%)	529 (5.2%)	60 (7.4%)	11,145 (7.9%)
**Wales**	3,807 (5.4%)	1,957 (4.2%)	75 (2.1%)	363 (3.6%)	329 (3.3%)	8 (1.0%)	6,539 (4.6%)
**Report working in patient-facing healthcare**							
**No**	69,449 (99.0%)	45,763 (97.2%)	3,560 (99.1%)	10,032 (98.3%)	9,751 (96.4%)	800 (99.1%)	139,355 (98.2%)
**Yes**	697 (1.0%)	1,302 (2.8%)	31 (0.9%)	175 (1.7%)	365 (3.6%)	7 (0.9%)	2,577 (1.8%)
**Report having a long-term health condition**							
**No**	52,348 (74.6%)	35,934 (76.3%)	3,131 (87.2%)	7,844 (76.8%)	8,349 (82.5%)	727 (90.1%)	108,333 (76.3%)
**Yes**	17,798 (25.4%)	11,131 (23.7%)	460 (12.8%)	2,363 (23.2%)	1,767 (17.5%)	80 (9.9%)	33,599 (23.7%)

Supplementary Table 1. Characteristics of participants with a single ChAdOx1, BNT162b2, or mRNA-1273 vaccination and at least one antibody measurement from 91 days before the first vaccination up to the second vaccination (if received) or breakthrough infections post-first vaccination. Continuous variables were compared using Kruskall-Wallis tests, and categorical variables were compared using one-sided Chi- squared tests.

		ChAdOx1			BNT162b2			mRNA-1273		
		Estimate	95% credible interval		Estimate	95% credible interval		Estimate	95% credible interval	
**Adjusted baseline**	Peak level (Intercept)	84	81	85	168	165	172	278	245	317
	IgG half-life (slope)	93	89	99	46	44	47	80	51	178
**Prior infection**	Peak level: No									
	IgG half-life: No									
	Change in peak level: Yes	**363**	**348**	**378**	**263**	**248**	**280**	**227**	**148**	**325**
	Change in half-life: Yes	-8	-18	3	**220**	**130**	**497**	**33**	**7**	**Not estimable**
**Age**	Peak level: 50 years									
	IgG half-life: 50 years									
	Change in peak level: per 10-years older	**-4**	**-5**	**-4**	**-28**	**-29**	**-27**	**-56**	**-67**	**-44**
	Change in half-life: per 10-years older	**6**	**3**	**9**	**2**	**1**	**3**	**32**	**10**	**Not estimable**
**Sex**	Peak level: Female									
	IgG half-life: Female									
	Change in peak level: Male	**-6**	**-7**	**-4**	**-13**	**-17**	**-9**	-32	-64	-3
	Change in half-life: Male	**-9**	**-15**	**-4**	0	-3	2	-21	-105	10
**Ethnicity**	Peak level: White									
	IgG half-life: White									
	Change in peak level: Non-white	**36**	**31**	**41**	**33**	**24**	**43**	-23	-78	40
	Change in half-life: Non-white	**-13**	**-22**	**-1**	-2	-6	3	20	-9	Not estimable
**Report having a long-term health condition**	Peak level: No									
	IgG half-life: No									
	Change in peak level: Yes	**-5**	**-7**	**-3**	**-17**	**-22**	**-13**	7	-38	60
	Change in half-life: Yes	-3	-10	4	0	-3	3	-31	-119	8

**Report working in patient-facing healthcare**	Peak level: No									
	IgG half-life: No									
	Change in peak level: Yes	6	-1	14	-11	-22	2	-54	-161	153
	Change in half-life: Yes	-18	-33	7	**25**	**12**	**49**		Not estimable	
**Deprivation percentile**	Peak level: 60 (median)									
	IgG half-life: 60 (median)									
	Change in peak level: per 10 percentile higher	0	-1	0	0	-1	1	3	-3	9
	Change in half-life: per 10 percentile higher	1	0	2	0	0	1	0	-15	13

Supplementary Table 2. Posterior median and 95% credible intervals for anti-spike IgG peak level (intercept) (BAU/mL) and half-life (slope) (days) in the multivariable models in 59,101 participants with first ChAdOx1 vaccination, 35,638 participants with first BNT162b2 vaccination, and 2,597 participants with first mRNA-1273 vaccination. The reference categories in the multivariable model are: no prior infection, 50-year-old, female, white ethnicity, not reporting a long-term health condition, not a healthcare worker, and deprivation percentile=60. Bold numbers indicate an effect not compatible with chance (95% credible interval excludes no effect). Model results are used to calculate estimations in **Table 1** and **Supplementary Table 3**. In mRNA-1273, some numbers are not estimable because antibody responses in certain groups were not estimated to decline.

			Peak level (BAU/mL)			Half-life (days)			Time to positivity threshold 23 BAU/mL (days)		
			One dose with prior infection	Two doses without prior infection	One dose without prior infection	One dose with prior infection	Two doses without prior infection	One dose without prior infection	One dose with prior infection	Two doses without prior infection	One dose without prior infection
**ChAd Ox1**	40	White female	471 (455-488)	157 (154-159)	89 (87-90)	81 (72-92)	81 (79-83)	88 (83-95)	381 (318-453)	246 (170-322)	200 (141-260)
		White male	439 (424-456)	152 (150-154)	83 (81-84)	74 (66-83)	82 (80-84)	80 (75-85)	342 (287-404)	244 (167-318)	175 (123-230)
		Non-white female	672 (640-707)	197 (192-203)	126 (121-131)	71 (62-83)	73 (70-77)	77 (68-88)	376 (314-448)	248 (181-318)	217 (165-275)
		Non-white male	627 (595-659)	191 (186-197)	118 (113-123)	66 (58-76)	74 (70-77)	70 (63-79)	342 (288-406)	246 (176-316)	194 (146-246)
	60	White female	424 (409-439)	162 (160-165)	80 (78-81)	90 (80-103)	80 (78-82)	99 (94-105)	407 (337-488)	246 (169-323)	206 (142-274)
		White male	395 (381-409)	157 (155-160)	74 (73-75)	81 (73-92)	80 (78-82)	89 (84-94)	362 (300-430)	244 (169-318)	178 (119-238)
		Non-white female	604 (574-636)	204 (198-211)	114 (109-118)	78 (67-94)	72 (69-76)	85 (75-99)	398 (329-484)	248 (182-317)	224 (165-288)
		Non-white male	563 (535-593)	198 (192-204)	106 (102-110)	72 (62-84)	73 (69-76)	77 (69-88)	359 (299-432)	245 (178-314)	198 (145-255)
	80	White female	381 (366-396)	168 (164-172)	72 (70-73)	102 (87-122)	79 (76-81)	113 (102-127)	440 (358-546)	247 (174-321)	214 (138-293)
		White male	355 (341-369)	163 (159-166)	67 (65-68)	91 (79-107)	79 (76-82)	100 (91-111)	386 (316-472)	243 (173-318)	181 (114-249)
		Non-white female	543 (513-574)	212 (204-219)	102 (97-107)	87 (73-109)	71 (68-75)	95 (81-116)	424 (341-534)	248 (181-315)	233 (166-311)
		Non-white male	506 (478-536)	205 (198-212)	95 (91-100)	79 (67-96)	71 (68-75)	86 (74-102)	379 (310-472)	247 (178-316)	204 (146-270)
**BNT16 2b2**	20	White female	741 (711-771)	1,243 (1,196-1,293)	289 (282-296)	150 (115-220)	51 (50-53)	40 (38-42)	780 (558-1,183)	323 (238-405)	175 (131-220)
		White male	682 (654-711)	1,066 (1,024-1,110)	266 (259-273)	147 (112-212)	51 (49-53)	40 (38-42)	745 (530-1,110)	308 (223-392)	169 (125-214)
		Non-white female	887 (836-942)	1,421 (1,331-1,520)	346 (329-364)	129 (94-207)	51 (48-54)	38 (35-43)	707 (489-1,141)	330 (244-416)	179 (136-224)
		Non-white male	817 (769-869)	1,218 (1,139-1,302)	319 (303-335)	126 (92-200)	50 (48-53)	38 (35-42)	679 (466-1,077)	315 (233-397)	173 (130-217)
	40	White female	517 (497-537)	1,057 (1,023-1,094)	202 (198-206)	212 (150-358)	52 (50-53)	44 (42-46)	979 (647-1,692)	312 (228-397)	164 (118-213)
		White male	476 (458-494)	906 (876-938)	186 (182-189)	206 (146-340)	51 (49-53)	43 (42-45)	924 (609-1,560)	297 (213-381)	159 (110-205)
		Non-white female	619 (583-657)	1,208 (1,134-1,290)	242 (230-254)	171 (114-340)	51 (48-54)	42 (38-46)	838 (528-1,672)	319 (237-405)	169 (122-217)
		Non-white male	570 (537-605)	1,035 (971-1,105)	222 (211-233)	167 (111-321)	51 (48-53)	41 (38-46)	798 (511-1,531)	305 (223-388)	163 (117-210)

	60	White female	361 (346-375)	898 (866-933)	141 (138-144)	356 (207-1205)	52 (50-53)	48 (45-50)	1,440 (780-4,837)	300 (216-384)	152 (100-206)
		White male	332 (319-346)	770 (742-798)	129 (127-132)	339 (199-1006)	51 (50-53)	47 (45-50)	1,338 (733-3,945)	285 (202-368)	146 (95-197)
		Non-white female	432 (406-459)	1,027 (960-1,099)	168 (160-177)	254 (143-1058)	51 (48-54)	45 (41-51)	1,102 (595-4,346)	307 (225-393)	158 (107-211)
		Non-white male	398 (374-423)	880 (822-941)	155 (147-163)	245 (139-897)	51 (48-54)	45 (40-50)	1,030 (572-3,682)	293 (211-377)	152 (102-203)
	80	White female	252 (240-264)	764 (728-801)	98 (95-101)	1120 (316-NE)	56 (52-60)	52 (49-56)	3,925 (1,064-NE)	289 (203-373)	138 (80-196)
		White male	231 (221-243)	655 (624-685)	90 (88-93)	952 (300-NE)	54 (50-59)	52 (49-56)	3,184 (948-NE)	274 (190-358)	131 (74-188)
		Non-white female	301 (282-322)	873 (809-942)	118 (111-124)	491 (190-NE)	49 (44-57)	49 (44-57)	1857 (696-NE)	297 (213-383)	144 (90-203)
		Non-white male	277 (260-296)	748 (694-805)	108 (102-114)	460 (183-NE)	48 (43-55)	49 (43-56)	1687 (664-NE)	283 (201-366)	138 (85-194)
**mRNA -1273**	20	White female	997 (812-1,232)		550 (475-637)	76 (39-1090)		41 (30-65)	437 (232-5,023)		216 (144-338)
		White male	881 (724-1,078)		486 (423-560)	57 (34-178)		35 (27-49)	325 (195-937)		180 (122-264)
		Non-white female	912 (686-1,223)		503 (392-649)	162 (42-NE)		58 (30-681)	894 (252-NE)		286 (155-2,764)
		Non-white male	806 (603-1,086)		445 (344-576)	94 (35-NE)		46 (27-174)	507 (206-NE)		224 (131-710)
	40	White female	634 (530-760)		349 (317-386)	191 (62-NE)		61 (46-91)	943 (313-NE)		267 (168-419)
		White male	560 (470-670)		309 (283-337)	102 (49-NE)		48 (39-62)	495 (237-NE)		207 (131-304)
		Non-white female	581 (443-762)		320 (255-401)	NE (68-NE)		106 (43-NE)	NE (361-NE)		427 (179-NE)
		Non-white male	513 (391-674)		282 (224-355)	363 (51-NE)		72 (36-NE)	1686 (262-NE)		287 (144-576)
	60	White female	403 (314-513)		222 (185-267)	NE (87-NE)		118 (55-NE)	NE (394-NE)		413 (180-NE)
		White male	356 (280-452)		196 (164-234)	514 (64-NE)		77 (45-297)	2105 (277-NE)		265 (135-928)
		Non-white female	368 (269-506)		203 (154-269)	NE (111-NE)		708 (57-NE)	NE (488-NE)		2252 (200-NE)
		Non-white male	326 (237-446)		179 (136-238)	NE (72-NE)		169 (45-NE)	NE (317-NE)		528 (145-NE)

Supplementary Table 3. Posterior predicted peak levels (BAU/mL), half-lives (days), and time from first/second dose to the positivity threshold (days) with 95% credible intervals in participants received one vaccination with prior infection, two vaccinations without prior infection, and one vaccination without prior infection, by vaccine type. Results were separated by age (20, 40, 60, 80-year-old), sex (female vs male) and ethnicity (white vs non-white). Estimations for two vaccinations without prior infection were based on our previous analysis[1]. NE: Not estimable, due to the antibody levels not declining in the posterior median or upper credible interval.

Scenario	Intercept	Slope	Correlation	Proportion of censored (%)	Coverage in intercept	Bias in intercept(95%CI)	Coverage in slope	Bias in slope (95%CI)	Coverage in correlation	Bias in correlation(95%CI)
1	8.5	-0.01	0	13-18	94%	0 (-0.02, 0.02)	94%	0.0001 (-0.0004, 0.0006)	94%	0 (-0.06, 0.08)
2	9.0	-0.01	0	38-44	96%	0 (-0.03, 0.04)	94%	0 (-0.0008, 0.001)	93%	0 (-0.08, 0.1)
3	8.8	-0.02	-0.2	12-16	97%	0 (-0.02, 0.02)	96%	0 (-0.0005, 0.0005)	97%	0 (-0.05, 0.06)
4	9.0	-0.01	-0.5	33-43	95%	0 (-0.03, 0.04)	95%	-0.0001 (-0.0006, 0.001)	98%	0 (-0.06, 0.05)
5	8.8	-0.02	0.5	17-22	93%	0 (-0.02, 0.03)	95%	0 (-0.0004, 0.0005)	96%	-0.01 (-0.05, 0.04)
6	9.0	-0.01	0.2	39-45	94%	0 (-0.02, 0.03)	96%	-0.0001 (-0.0006, 0.0006)	95%	0.01 (-0.06, 0.05)

Supplementary Table 4. Simulation results on validating the Bayesian interval-censored linear mixed model is robust to the upper quantification limit. Three scenarios were modelled: negative correlation, no correlation, and positive correlation between intercept and slope. Within each scenario, two parameter sets were chosen to line up with the different proportion of censored measurements (the proportion of censored data was 11% for ChAdOx1, 19% for BNT162b2, and 38% for mRNA-1273). 1000 samples were used each model and 100 repetitions of each simulation were performed. Simulations results showed good coverage and no/negligible bias, indicating the model is robust to the upper limit of detection of the assay.

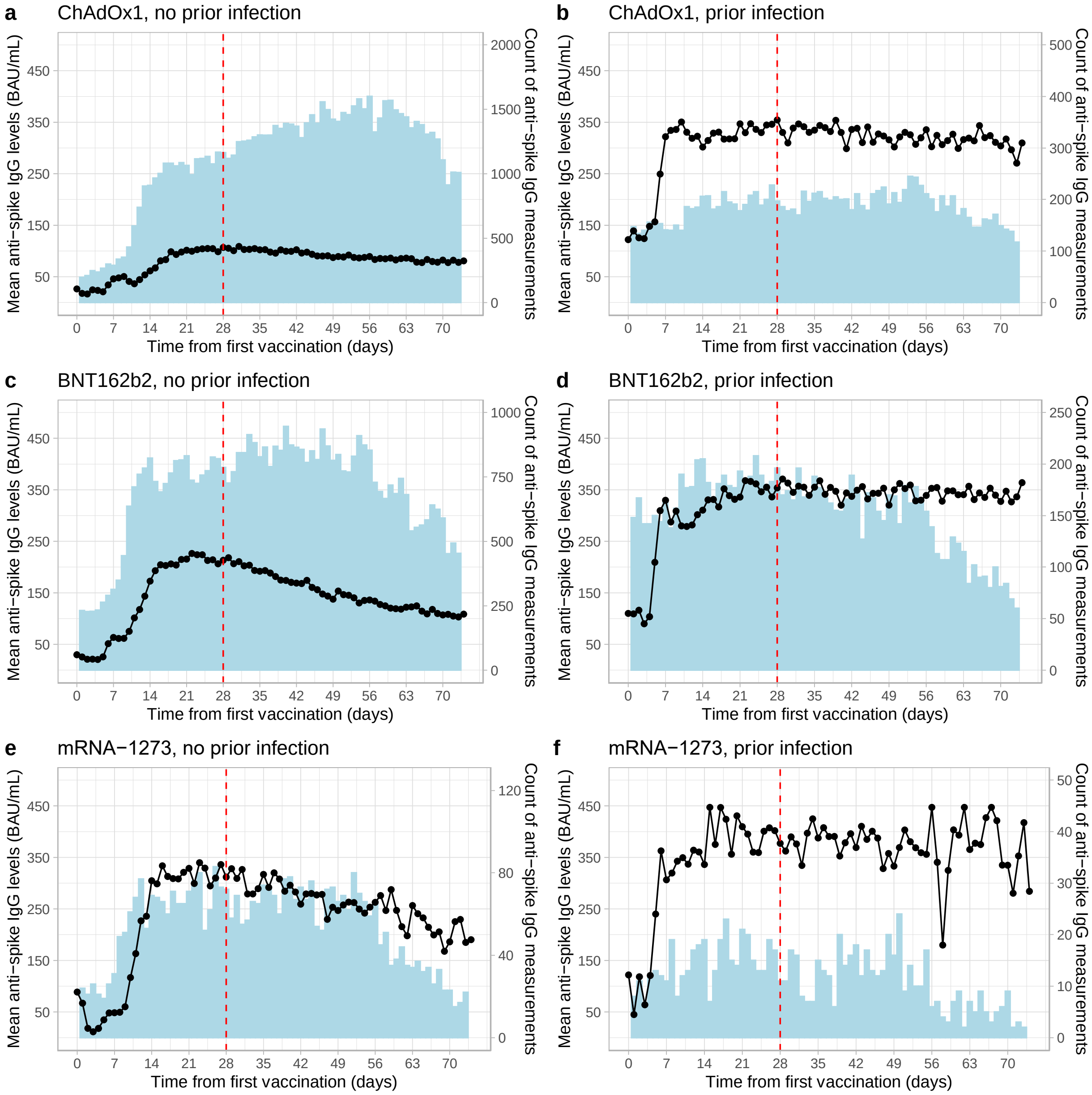

Supplementary Fig. 1. Mean anti-spike IgG levels (BAU/mL) and count of anti-spike IgG measurements by day after the first vaccination. Panels are separated by vaccines (ChAdOx1, BNT162b2, mRNA-1273) and prior infection status. Red dotted lines represent the time of the 'peak level', which is 28 days. Values truncated at 450 BAU/mL counted as =450 BAU/mL.

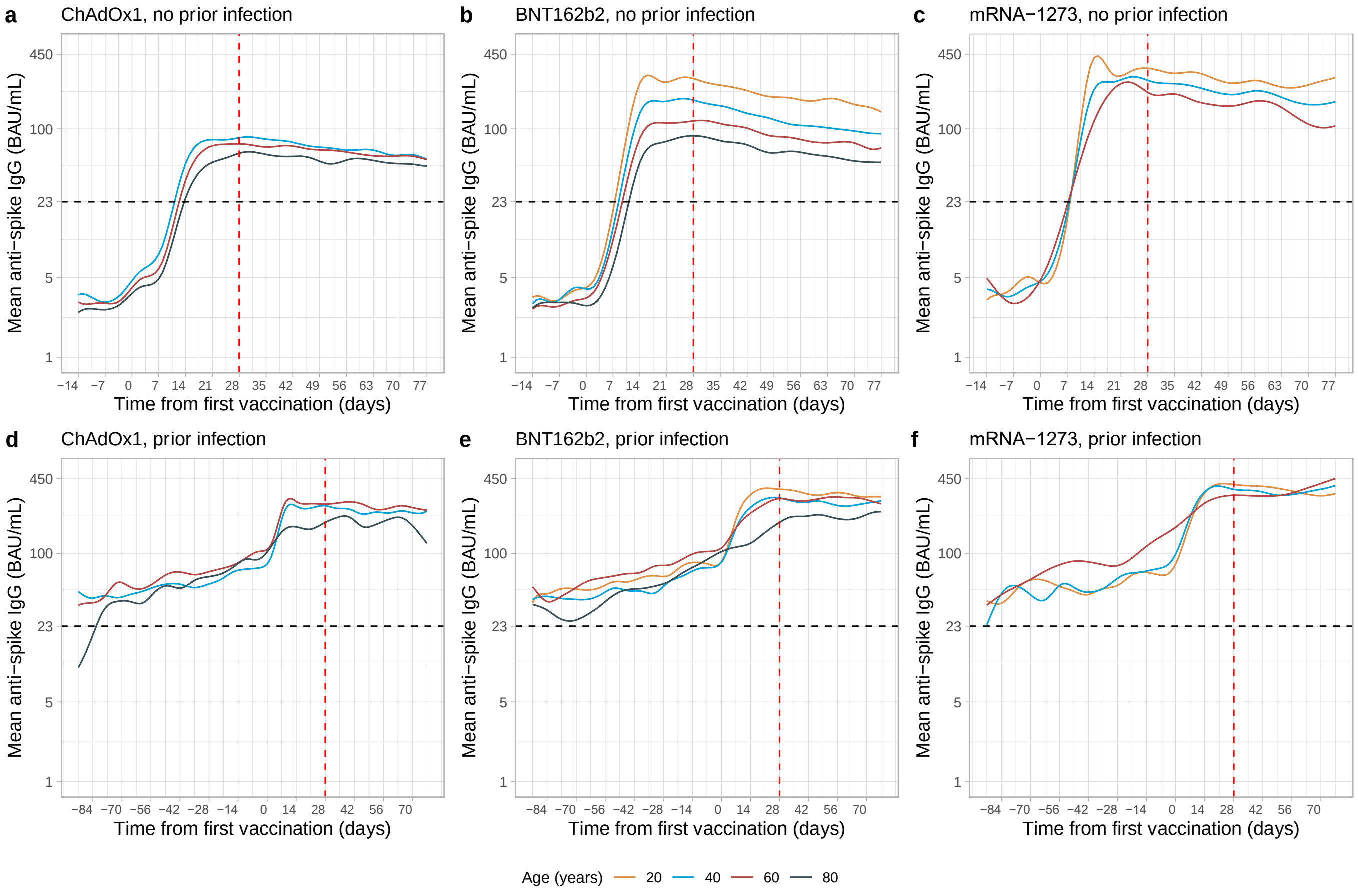

Supplementary Fig. 2. Mean anti-spike IgG levels (95% CI) by time from first vaccination by age, vaccine type, and prior infection status. a, No prior infection and received one ChAdOx1 vaccination, N=70,146. **b**, No prior infection and received one BNT162b2 vaccination, N=47,065. **c**, No prior infection and received one mRNA-1273 vaccination, N=3,591. **d**, With prior infection and received one ChAdOx1 vaccination, N=10,207. **e**, With prior infection and received one BNT162b2 vaccination, N=10,116. **f**, With prior infection and received one mRNA-1273 vaccination, N=807. Predicted levels are plotted on a log 10 scale. Black dotted line indicates the threshold of IgG positivity (23 BAU/mL). Red dotted lines represent the time of the 'peak level', which is 28 days. Line colour indicates response predicted for ages 20, 40, 60, and 80 years. The 95% CIs are calculated by prediction ± 1.96 × standard error of the prediction. Values truncated at 450 BAU/mL counted as =450 BAU/mL.

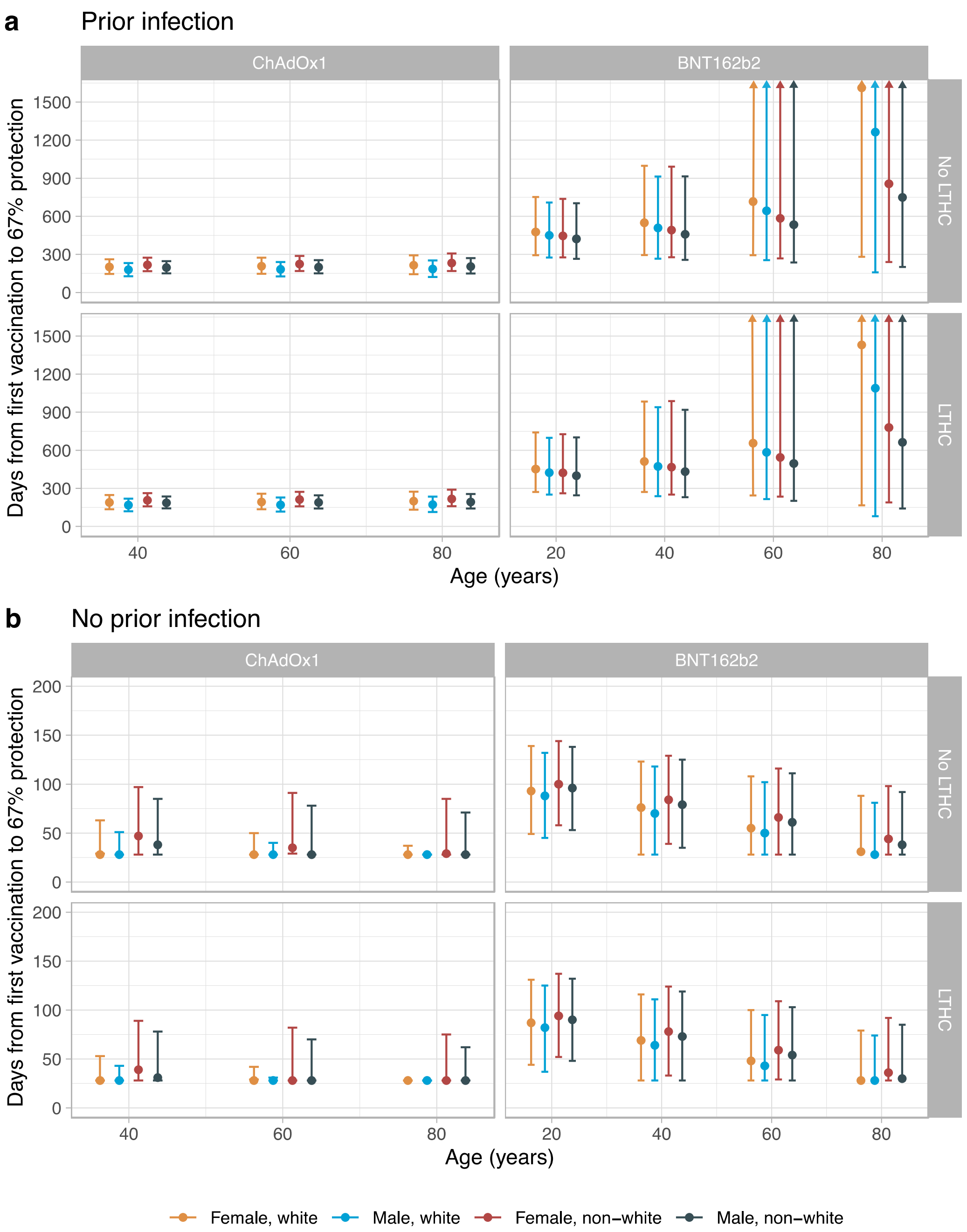

Supplementary Fig. 3. Posterior predicted mean days (95% credible interval) from the first vaccination to the threshold level associated with 67% protection (107 BAU/mL for ChAdOx1 and 94 BAU/mL for BNT162b2). a, in those with evidence of prior infection (n=7,191, 5,523 for ChAdOx1, BNT162b2, respectively)**. b**, in those without evidence of prior infection (n=51,910, 30,115 for ChAdOx1, BNT162b2, respectively). Estimates were separated by age (predicted at 20, 40, 60, and 80-year-old), sex, ethnicity, long-term health condition (LTHC), and vaccine type. The correlates of protection were reported in our previous study on two vaccine doses[1]. For ChAdOx1, the 20-year-old group is not plotted because the vast majority of those receiving ChAdOx1 were ≥40 years. mRNA-1273 is not plotted because we did not have enough data to estimate its correlate of protection.

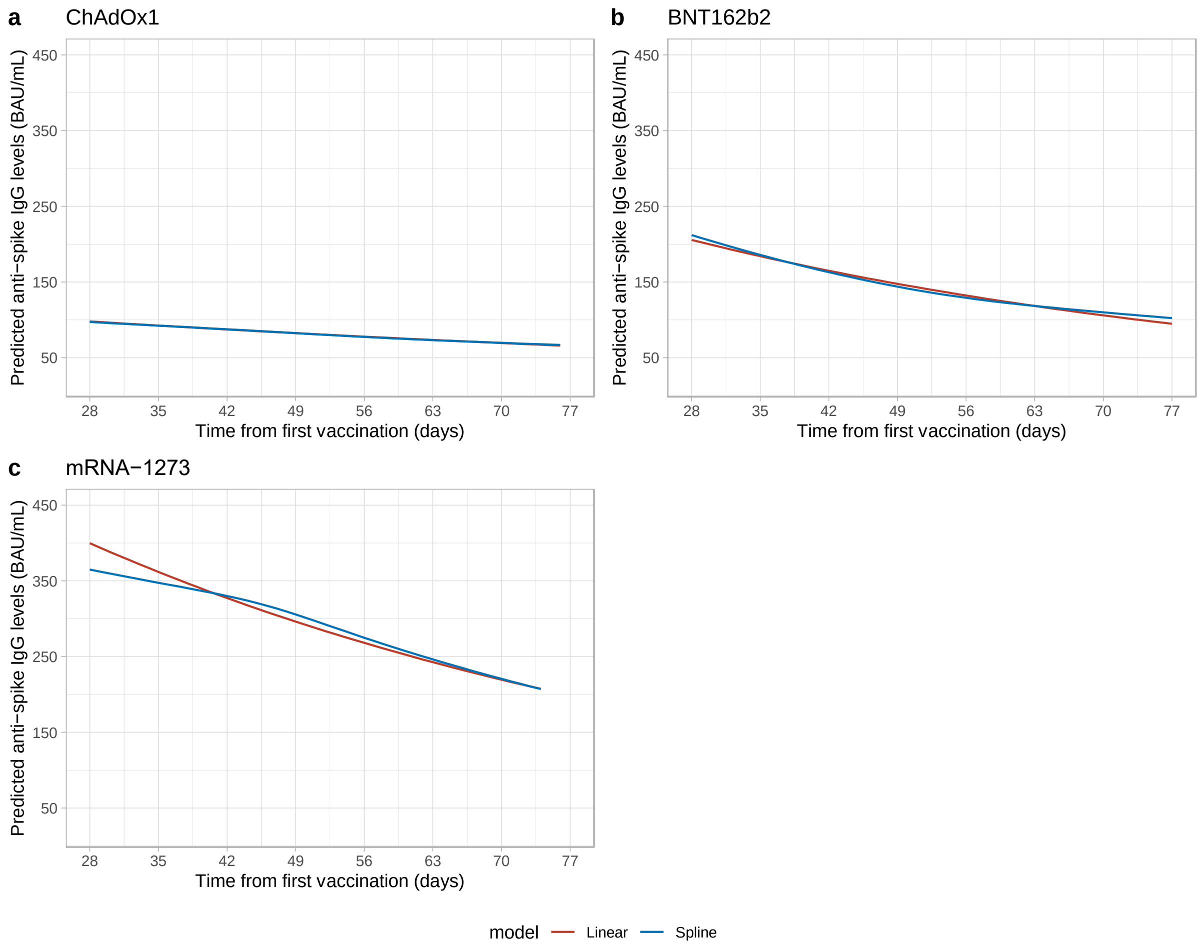

Supplementary Fig. 4. Comparison of linear exponential model with spline-based model in examining non-linearity of antibody decline. The estimated trajectory from the spline model (with 4 knots placed at 10th, 33rd, 67th, and 90th of observed time points) is similar with the linear exponential model for all three vaccines, indicating that there was no evidence of antibody decline flattening.

Reference

1. Wei, J. *et al.* Antibody responses and correlates of protection in the general population after two doses of the ChAdOx1 or BNT162b2 vaccines. *Nature Medicine 2022* 1–11 (2022) doi:10.1038/s41591-022-01721-6.
