# Supplementary material for: SARS-CoV-2 antibody trajectories after a single COVID-19 vaccination with and without prior infection
Source: Nat Commun. 2022 Jun 29;13:3748. doi: 10.1038/s41467-022-31495-x (PMC9243074; doi:10.1038/s41467-022-31495-x)
Supplement: Supplementary file 3 — Reporting Summary [file 41467_2022_31495_MOESM3_ESM.pdf]

nature portfolio

Corresponding author(s): Koen Pouwels

Last updated by author(s): May 24, 2022

Reporting Summary

Nature Portfolio wishes to improve the reproducibility of the work that we publish. This form provides structure for consistency and transparency in reporting. For further information on Nature Portfolio policies, see our Editorial Policies and the Editorial Policy Checklist.

Statistics

For all statistical analyses, confirm that the following items are present in the figure legend, table legend, main text, or Methods section.

n/a	Confirmed	
☐	☒	The exact sample size (*n*) for each experimental group/condition, given as a discrete number and unit of measurement
☐	☒	A statement on whether measurements were taken from distinct samples or whether the same sample was measured repeatedly
☐	☒	The statistical test(s) used AND whether they are one- or two-sided<br>*Only common tests should be described solely by name; describe more complex techniques in the Methods section.*
☐	☒	A description of all covariates tested
☐	☒	A description of any assumptions or corrections, such as tests of normality and adjustment for multiple comparisons
☐	☒	A full description of the statistical parameters including central tendency (e.g. means) or other basic estimates (e.g. regression coefficient) AND variation (e.g. standard deviation) or associated estimates of uncertainty (e.g. confidence intervals)
☐	☒	For null hypothesis testing, the test statistic (e.g. *F*, *t*, *r*) with confidence intervals, effect sizes, degrees of freedom and *P* value noted<br>*Give P values as exact values whenever suitable.*
☐	☒	For Bayesian analysis, information on the choice of priors and Markov chain Monte Carlo settings
☒	☐	For hierarchical and complex designs, identification of the appropriate level for tests and full reporting of outcomes
☒	☐	Estimates of effect sizes (e.g. Cohen's *d*, Pearson's *r*), indicating how they were calculated

Our web collection on statistics for biologists contains articles on many of the points above.

Software and code

Policy information about availability of computer code

Data collection	The UK's Office for National Statistics (ONS) COVID-19 Infection Survey (CIS) randomly selects private households on a continuous basis from address lists and previous surveys. Individuals were surveyed on their socio-demographic characteristics, behaviours, and vaccination status. Combined nose and throat swabs were taken from all consenting household members for SARS-CoV-2 PCR testing. For a random 10-20% of households, individuals ≥16 years were invited to provide blood samples monthly for serological testing. Household members of participants who tested positive were also invited to provide blood monthly for follow-up visits.<br>De-identified study data were accessed through the Office for National Statistics (ONS) Secure Research Service (SRS). The data available in SRS were prepared and processed using Stata MP 16.
Data analysis	PCR outputs were analysed using UgenTec FastFinder 3.300.5, with an assay-specific algorithm and decision mechanism that allows conversion of amplification assay raw data into test results with minimal manual intervention.<br>All analyses were performed in R 4.1 using the following packages: tidyverse (version 1.3.1), brms (version 2.15.0), arsenal (version 3.4.0), cowplot (version 1.1.1), bayesplot (version 1.8.1), and tidybayes (version 3.0.1). A copy of the analysis code is available at https://github.com/jiaweioxford/COVID19_antibody_response_first_dose.

For manuscripts utilizing custom algorithms or software that are central to the research but not yet described in published literature, software must be made available to editors and reviewers. We strongly encourage code deposition in a community repository (e.g. GitHub). See the Nature Portfolio guidelines for submitting code & software for further information.

Data

Policy information about availability of data

All manuscripts must include a data availability statement. This statement should provide the following information, where applicable:

- Accession codes, unique identifiers, or web links for publicly available datasets
- A description of any restrictions on data availability
- For clinical datasets or third party data, please ensure that the statement adheres to our policy

De-identified study data are available for access by accredited researchers in the ONS Secure Research Service (SRS) for accredited research purpose under part 5, chapter 5 of the Digital Economy Act 2017. Individuals can apply to be an accredited researcher using the short form on https://researchaccreditationservice.ons.gov.uk/ons/ONS_registration.ofml. Accreditation requires completion of a short free course on accessing the SRS. To request access to data in the SRS, researchers must submit a research project application for accreditation in the Research Accreditation Service (RAS). Research project applications are considered by the project team and the Research Accreditation Panel (RAP) established by the UK Statistics Authority at regular meetings. Project application example guidance and an exemplar of a research project application are available. A complete record of accredited researchers and their projects is published on the UK Statistics Authority website to ensure transparency of access to research data. For further information about accreditation, contact Research.Support@ons.gov.uk or visit the SRS website.

Field-specific reporting

Please select the one below that is the best fit for your research. If you are not sure, read the appropriate sections before making your selection.

☒ Life sciences ☐ Behavioural & social sciences ☐ Ecological, evolutionary & environmental sciences

For a reference copy of the document with all sections, see nature.com/documents/nr-reporting-summary-flat.pdf

Life sciences study design

All studies must disclose on these points even when the disclosure is negative.

Sample size	141,932 participants aged ≥16 years from the general population of the United Kingdom who received at least a single vaccination with ChAdOx1 or BNT162b2 or mRNA-1273 with antibody measurements from 8th December 2020 until 18th October 2021 were included. All available data were used for the current study, with the timing of the analysis determined by the duration of follow up available, rather than sample size given the number of participants in the study.
Data exclusions	To estimate antibody waning, we excluded a small number of participants who were considered as non-responders after the first dose, defined as all antibody measurements being <16 BAU/mL and having at least one antibody measurement 21 days after the first dose (N=4,488 excluded for ChAdOx1, N=1,450 excluded for BNT162b2, N=17 excluded for mRNA-1273).
Replication	All measurements and analytical assays were undertaken once given the scale of the study and cost limitations with 100,000s of assays performed. Serially collected samples from the same participants included demonstrate reproducibility over time. The statistical analyses have been successfully replicated by two individuals.
Randomization	Recruitment randomised - we used data from the UK's Office for National Statistics (ONS) COVID-19 Infection Survey (CIS) (ISRCTN21086382) which randomly selects private households on a continuous basis from address lists and previous surveys conducted by the ONS or the Northern Ireland Statistics and Research Agency to provide a representative sample across the four countries comprising the UK (England, Wales, Northern Ireland, Scotland). No intervention.
Blinding	Not done. This was an observational study with no interventions. Results were returned to participants to support their involvement, but this would not be expected to impact the study findings.

Reporting for specific materials, systems and methods

We require information from authors about some types of materials, experimental systems and methods used in many studies. Here, indicate whether each material, system or method listed is relevant to your study. If you are not sure if a list item applies to your research, read the appropriate section before selecting a response.

Materials & experimental systems

n/a	Involved in the study	
☐	☒	Antibodies
☒	☐	Eukaryotic cell lines
☒	☐	Palaeontology and archaeology
☒	☐	Animals and other organisms
☐	☒	Human research participants
☐	☐	Clinical data
☒	☐	Dual use research of concern

Methods

n/a	Involved in the study	
☒	☐	ChIP-seq
☒	☐	Flow cytometry
☒	☐	MRI-based neuroimaging

Antibodies

Antibodies used

The calibrant (mAb45) provided as part of the Thermo Fisher OmniPATH 384 Combi SARS-CoV-2 IgG ELISA kit is a monoclonal antibody. It tis available as part of the test kit. CR3022 and mAb269 were produced at the University of Oxford, details on availability can be provided by the authors on request.
We calibrated the results of the Thermo Fisher OmniPATH assay into WHO international units (binding antibody unit, BAU/mL) using serial dilutions of National Institute for Biological Standards and Control (NIBSC) Working Standard 21/234.

Validation

Details of the validation of the Thermo Fisher OmniPATH 384 Combi SARS-CoV-2 IgG ELISA kit are provided in the manufacturer's instructions for use. The assay has also been validated in a head-to-head comparison of similar assays (https://doi.org/10.1016/S1473-3099(20)30634-4), where the sensitivity was 99.1% (95%CI 97.8-99.7) and specificity was 99.0% (98.1-99.5). The assay calibrant, mAb45, is described in https://doi.org/10.1016/j.cell.2021.02.032.

The CR3022 monoclonal antibody is described and validated in https://doi.org/10.1016/j.chom.2020.06.010. The mAb269 is described and validated in https://doi.org/10.1016/j.cell.2021.02.033.

Further validation of the antibodies used was performed by comparing serial dilution series of these antibodies on the test platform, ensuring a sigmoidal dose response that these saturated at the upper limit of detection. Reproduibility between batches and stability over time was also assessed using the same method. Comparison of the performance of these antibodies on a commercial anti-S antibody detection platform are provided in https://doi.org/10.1016/j.cmi.2021.05.041

Human research participants

Policy information about studies involving human research participants

Population characteristics

The median (IQR) age was 50 (37-63) years, 75,593 (53.3%) were female, and 131,365 (92.6%) reported white ethnicity. 2,577 (1.8%) reported working in patient-facing healthcare, and 33,599 (23.7%) having a long-term health condition. 70,146 (49.4%), 47,065 (33.1%) and 3,591 (2.5%) participants without evidence of prior infection received a single dose of ChAdOx1 or BNT162b2 or mRNA-1273, as did 10,207 (7.2%) , 10,116 (7.1%) and 807 (0.6 %) with evidence of prior infection, respectively.

Recruitment

The Office for National Statistics (ONS) COVID-19 Infection Survey (CIS) is a large household survey with longitudinal follow-up (ISRCTN21086382, https://www.ndm.ox.ac.uk/covid-19/covid-19-infection-survey/protocol-and-information-sheets) (details in20). The study received ethical approval from the South Central Berkshire B Research Ethics Committee (20/SC/0195). Private households are randomly selected on a continuous basis from address lists and previous surveys to provide a representative sample across the UK. Following verbal agreement to participate, a study worker visited each selected household to take written informed consent for individuals aged 2 years and over. Parents or carers provided consent for those aged 2-15 years; those aged 10-15 years also provided written assent. All participants who completed the enrolment visit was offered a £50 voucher, and one £25 voucher for each further visit. For the current analysis we only included individuals aged 16 years and over.

Nevertheless a certain degree of non-response is inevitable. While certain factors might drive non-response to invitations to participate, adjustment for covariates that may influence selection into the sample ensures that estimates of relative effects are not biased by factors that both influence selection into the sample and the risk of the outcome (model-based inference). Factors that were included in the model are listed below. We cannot exclude the possibility that other unmeasured factors that could influence self-selection into the survey and are not strongly associated with factors already included in the model could bias the results.

When fitting fitting the Bayesian linear mixed models, we adjust for age, sex, ethnicity, reported long-term health conditions, an indicator for healthcare workers, and deprivation percentile as covariates in the multivariable mode.

Ethics oversight

The study received ethical approval from the South Central Berkshire B Research Ethics Committee (20/SC/0195).

Note that full information on the approval of the study protocol must also be provided in the manuscript.

Clinical data

Policy information about clinical studies

All manuscripts should comply with the ICMJE guidelines for publication of clinical research and a completed CONSORT checklist must be included with all submissions.

Clinical trial registration	ISRCTN21086382
Study protocol	https://www.ndm.ox.ac.uk/covid-19/covid-19-infection-survey/protocol-and-information-sheets
Data collection	The Office for National Statistics (ONS) COVID-19 Infection Survey (CIS) is a large household survey with longitudinal follow-up (ISRCTN21086382, https://www.ndm.ox.ac.uk/covid-19/covid-19-infection-survey/protocol-and-information-sheets) (details in20). Following verbal agreement to participate, a study worker visited each selected household to take written informed consent for individuals aged 2 years and over. Parents or carers provided consent for those aged 2-15 years; those aged 10-15 years also provided written assent. For the current analysis we only included individuals aged 16 years and over.<br>Individuals were asked about demographics, behaviours, work, and vaccination uptake (https://www.ndm.ox.ac.uk/covid-19/covid-19-infection-survey/case-record-forms). At the first visit, participants were asked for (optional) consent for follow-up visits every week for the next month, then monthly for 12 months from enrolment. At each visit, enrolled household members provided a nose and throat self-swab following instructions from the study worker, which is comparable to or even more sensitive than swabs done by healthcare workers. From a random 10-20% of households, those 16 years or older were invited to provide blood monthly for antibody testing. From April 2021, additional participants were invited to provide blood samples monthly to assess vaccine responses, targeting 150,000 antibody tests per month, based on a combination of random selection and prioritization of individuals in the study for the longest period (independent of test results, vaccination or previous positive PCR tests). Throughout, individuals with a positive swab test and their household members were also invited to provide blood monthly for follow-up visits after this.
Outcomes	SARS-CoV-2 antibody levels were measured using an ELISA detecting anti-trimeric spike IgG developed by the University of Oxford (Thermo Fisher OmniPATH 384 Combi SARS-CoV-2 IgG ELISA).<br>Normalised results are reported in ng/ml of mAb45 monoclonal antibody equivalents. Before 26 February 2021, the assay used fluorescence detection as previously described, with a positivity threshold of 8 million units validated on banks of known SARS-CoV-2 positive and negative samples. After this, it used a commercialised CE-marked version of the assay, the Thermo Fisher OmniPATH 384 Combi SARS-CoV-2 IgG ELISA (Thermo Fisher Scientific), with the same antigen and colorimetric detection. mAb45 is the manufacturer-provided monoclonal antibody calibrant for this quantitative assay. To allow conversion of fluorometrically determined values in arbitrary units, we compared 3,840 samples which were run in parallel on both systems. A piece-wise linear regression was used to generate the following conversion formula:<br>(1) log10(mAb45 units) = 0.221738 + 1.751889e-07*fluorescence_units + 5.416675e-07*(fluorescence_units>9190310)*(fluorescence_units-9190310)<br><br>We calibrated the results of the Thermo Fisher OmniPATH assay into WHO international units (binding antibody unit, BAU/mL) using serial dilutions of National Institute for Biological Standards and Control (NIBSC) Working Standard 21/234. The NIBSC 21/234 Working Standard has been previously calibrated against the WHO International Standard for anti-SARS-CoV-2 immunoglobulin (NIBSC code 20/136), with anti-spike IgG potency of 832 BAU/mL (95%CI 746-929). We generated 2-fold dilutions of 21/234 between 1:400 and 1:8000 from three separate batches on three separate days. Results from a total of 63 diluted samples were merged and a linear regression model fitted constrained to have an intercept of zero to convert mAB45 units in ng/ml for samples diluted at 1:50 to BAU/mL:<br>BAU/mL = 0.559 * [mAb45 concentration in ng/mL at 1:50]<br>23 BAU/mL was used as the threshold for an IgG positive or negative result (corresponding to the 8 million units with fluorescence detection). Given the lower and upper limits of the assay, measurements <1 BAU/mL (2533 observations, 0.8%) and >450 BAU/mL (28,086 observations, 8.4%) were truncated at 1 and 450 BAU/mL, respectively.<br><br>For the models evaluating antibody responses, the outcome was right-censored at 450 BAU/mL reflecting truncation of IgG values at the upper limit of quantification (i.e. all measurements truncated to 450 BAU/mL were considered to be >450 BAU/mL in analyses).
